# A novel pathogenic m.4412G>A *MT-TM* mitochondrial DNA variant associated with childhood-onset seizures, myopathy and bilateral basal ganglia changes

**DOI:** 10.1016/j.mito.2019.04.007

**Published:** 2019-07

**Authors:** Albert Z. Lim, Emma L. Blakely, Karen Baty, Langping He, Sila Hopton, Gavin Falkous, Kenneth McWilliam, Alison Cozens, Robert McFarland, Robert W. Taylor

**Affiliations:** a Wellcome Centre for Mitochondrial Research, Institute of Neuroscience, Newcastle University, Newcastle upon Tyne NE2 4HH, UK; b NHS Highly Specialised Service for Rare Mitochondrial Disorders of Adults and Children, Newcastle upon Tyne Hospitals NHS Foundation Trust, Newcastle upon Tyne NE2 4HH, UK; c Department of Paediatric Neurology, Royal Hospital for Sick Children, Edinburgh EH9 1LF, UK; d Inherited Metabolic Disorders Scotland, NHS National Services Scotland, Glasgow G2 6QE, UK

**Keywords:** Mitochondrial disease, Myopathy, Seizures, mtDNA variant, Basal ganglia changes, *MTTM*

## Abstract

Mitochondrial DNA variants in the *MT-TM* (mt-tRNA^Met^) gene are rare, typically associated with myopathic phenotypes. We identified a novel *MT-TM* variant resulting in prolonged seizures with childhood-onset myopathy, retinopathy, short stature and elevated CSF lactate associated with bilateral basal ganglia changes on neuroimaging. Muscle biopsy confirmed multiple respiratory chain deficiencies and focal cytochrome *c* oxidase (COX) histochemical abnormalities. Next-generation sequencing of the mitochondrial genome revealed a novel m.4412G>A variant at high heteroplasmy levels in muscle that fulfils all accepted criteria for pathogenicity including segregation within single muscle fibres, thus broadening the genotypic and phenotypic landscape of mitochondrial tRNA-related disease.

## 1 Introduction

The circular mitochondrial DNA (mt-DNA) contains 13 genes that encode essential subunits of the oxidative phosphorylation complexes, 2 rRNA genes and 22 tRNA genes(Schon et al., 2012). The mitochondrial tRNA (mt-tRNA) genes, which contributes <10% of the total coding sequence of the mitochondrial genome, are known as pathogenic hotspots because they are responsible for more than half of mtDNA-related diseases(Taylor and Turnbull, 2005; Yarham et al., 2010). The point mutations in them typically cause a loss of its stability leading to defective mitochondrial translation and combined respiratory chain deficiency(Blakely et al., 2013). Although these mt-tRNA mutations are responsible for most of the mt-DNA-related diseases in adults phenotypes(Gorman et al., 2015) such as mitochondrial encephalopathy, lactic acidosis, and stroke-like episodes (MELAS)(Goto et al., 1990) and myoclonic epilepsy with ragged-red fibres (MERRF)(Shoffner et al., 1990), they are very uncommon in children(Darin et al., 2001; Scaglia et al., 2004). Furthermore, amongst the mutations in mt-tRNA genes, those in mt-tRNA^Met^ are rare with clinical cases associated with just a small number of reported variants (m.4403G>A, m.4409T>C, m.4440G>A, m.4437C>T and m.4450G>A(Vissing et al., 1998; Lombes et al., 1998; Olsen et al., 2003; Peverelli et al., 2014; Scarpelli et al., 2018; Tang et al., 2013; Kuwajima et al., 2019; Born et al., 2015)). We report a novel m.4412G>A variant in the *MT-TM* gene with high levels of heteroplasmy in skeletal muscle in a young female with disease onset at age 10 years and our work to confirm its pathogenicity.

## 2 Materials and methods

### 2.1 Patient and clinical investigations

Our patient was identified following referral to the UK NHS Highly Specialised Service for Rare Mitochondrial Disease in Newcastle upon Tyne, UK. Informed consent from the patient's parents was obtained for the publication of relevant clinical information including photographs and all clinical investigations were carried out in accordance to the Declaration of Helsinki.

### 2.2 Histochemical and biochemical analyses

Standard histological (Hematoxylin & Eosin, modified Gomori trichrome staining) and histochemical (cytochrome *c* oxidase (COX), succinate dehydrogenase (SDH) and sequential COX-SDH reactions) analyses were performed on fresh frozen skeletal muscle sections. Mitochondrial respiratory chain complex activities were evaluated in a skeletal muscle homogenate and expressed relative to the activity of the matrix enzyme marker, citrate synthase, as described(Kirby et al., 2007). Additionally, the mitochondrial oxidative phosphorylation (OXPHOS) function was assessed using a quadruple immunohistochemical assay of Complex I (NDUFB8), complex IV (COX-1) and porin (mitochondrial mass marker) immunoreactivity as previously reported(Rocha et al., 2015).

### 2.3 Molecular genetics

Total DNA was extracted from available tissues (skeletal muscle, circulating lymphocytes, urinary sediment and buccal epithelial cells) using standard methodologies. The entire mitochondrial genome was amplified in two overlapping fragments by long-range PCR of muscle DNA and analysed by Next Generation sequencing (NGS) using an Ion Torrent™ Personal Genome Machine (PGM) platform (Thermo Fisher Scientific). Sequences were aligned to the revised Cambridge sequence (GenBank Accession number NC_012920.1) for human mtDNA(Andrews et al., 1999). Almost all of the mitochondrial genome (99.99%) was covered at a read depth of 200× with a detection sensitivity of ≥5% heteroplasmy for single base substitutions. Data analysis was performed in Torrent Suite v5.0.4 using Variant Caller v5.0.4.0 and Coverage Analysis v5.0.4.0.

### 2.4 Assessment of mutation load by quantitative pyrosequencing

A novel m.4412G>A variant identified following mtDNA sequencing was further assessed by a quantitative pyrosequencing assay using mutation-specific primers (details available on request). mtDNA heteroplasmy levels were determined in DNA samples from the patient and her mother, as well as individual laser-captured COX-deficient and COX-positive muscle fibres. Quantification of mtDNA mutation loads was achieved using Pyromark Q24 software.

## 3 Results

### 3.1 Clinical case report

A 10-year-old female Caucasian, born of non-consanguineous parents, had presented with generalised status epilepticus for more than 2 h, thus requiring intubation and admission to a paediatric intensive care unit. Prior to the seizure, she had been well but later recalled that she had strenuous physical activity at an outdoor pursuit centre. She was extubated after 24 h and, was discharged home after 3 days without any apparent neurological sequelae. One year later, at age 11 years, she had another prolonged generalised tonic-clonic seizure that terminated with benzodiazepines and intravenous phenytoin.

Apart from recurrent history of prolonged seizures, she had complained of fatigue and reduced exercise tolerance since early childhood. Her mother recalled that she had difficulties keeping up her peers around the age 2 years and had tendencies to avoid physical exertion. For long distances, she required the wheelchair to avoid excessive tiredness and myalgia. Since the onset of her first seizure, she had gradually struggled with her gait and felt significantly unsteady around age 12 years.

Another medical observation was her short stature and poor weight gain since age 5 years. After an unsuccessful trial of nasogastric tube feeding, she eventually had percutaneous enteral gastrostomy (PEG) to improve her growth. Her birth history was unremarkable and her early neurodevelopment was entirely appropriate, but she had been struggling academically following the onset of her seizures. Both parents were healthy and her other two siblings were clinically unaffected.

**Fig. 1:**
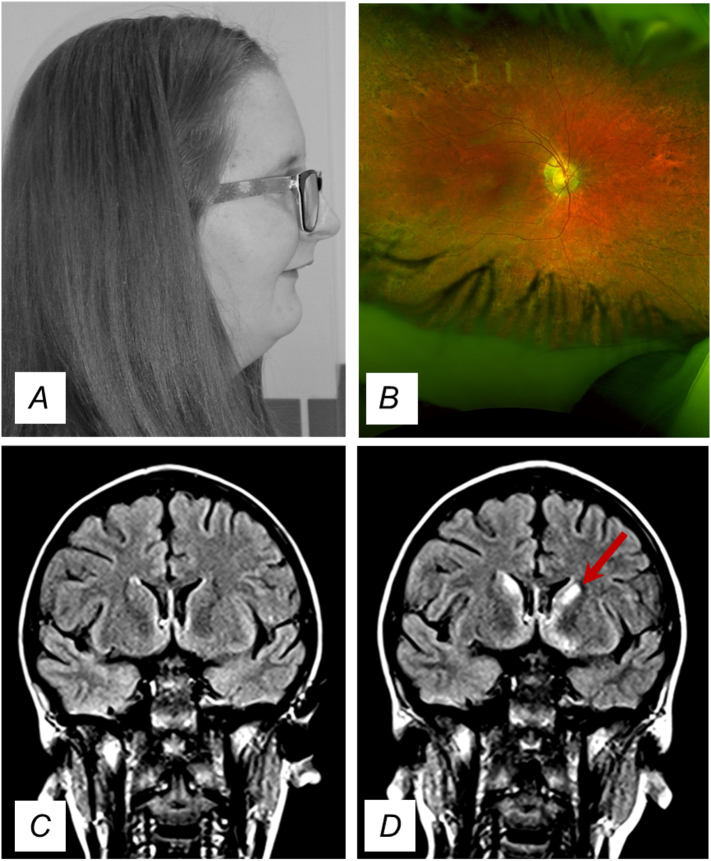
Clinical features. (A) Clinical photography of the patient (aged 16 years) showing an underdeveloped lower jaw. (B) Retinal photography revealed pigmentary changes on her retina. (C) MRI T2 FLAIR coronal view performed at the age of 12 years showed normal T2 signal in the caudate nucleus. (D) Similar view performed at the age of 13 years showed high T2 signal in the caudate nucleus bilaterally; the red arrow indicates this abnormality on the left. This MRI signal change is likely to have developed during the 1 year interval between the two scans and coincides with a deterioration in her gait. (For interpretation of the references to colour in this figure legend, the reader is referred to the web version of this article.)

On examination, both her height (1.44 m) and her head circumference were <0.4th centile. She was 20 cm shorter than her predicted mid-parental height of 50th centile (1.64 m). Whilst, her weight had fallen from 75th centile at birth to the current 0.4th-2nd centile at age 16 years. From the neurological perspective, her gait was broad-based and her tone was low with proximal weakness. No other significant bedside examination findings were observed apart from mild degree of underdeveloped lower jaw (Fig. 1A) and a PEG tube. Fundoscopic examination of her eyes showed evidence of retinitis pigmentosa (Fig. 1B).

### 3.2 Clinical investigations

Her lactate levels were high in both cerebrospinal fluid, (6.9 mmol/L), and serum, (7.5 mmol/L) as well as mildly elevated creatine kinase at 316 units/L (normal 25–200 units/L). During her seizure episodes, her CSF lactate peaked at 17.2 mmol/L. All her other clinical laboratory results including inflammatory markers, kidney function, thyroid function, neuro-immunology screen, caeruloplasmin, vitamin levels, free fatty acids, acylcarnithine profile, ammonia, amino acids, urinary organic acids, autoimmune screen and toxicology screen were normal. Her initial MRI brain scan at her first seizure presentation age 10 years and her interim scan at age 12 years showed no significant abnormalities (Fig. 1C) but her subsequent MRI brain imaging at age 13 years showed high T2 signal in the caudate heads bilaterally (more prominent on left) (Fig. 1D). In between these brain MRI scans, her gait and coordination deteriorated significantly. Her cardiac review demonstrated no evidence of cardiomyopathy or conduction abnormalities. The electroencephalography (EEG) following her prolonged seizure age 10 years showed non-specific encephalopathic changes. Her subsequent inter-ictal EEG at age 11 years showed normal background activity with a photoparoxysmal response at 22 Hz. There was frontal epileptiform activity during drowsiness, without clinical concomitant.

### 3.3 Histochemical and biochemical analyses

**Fig. 2:**
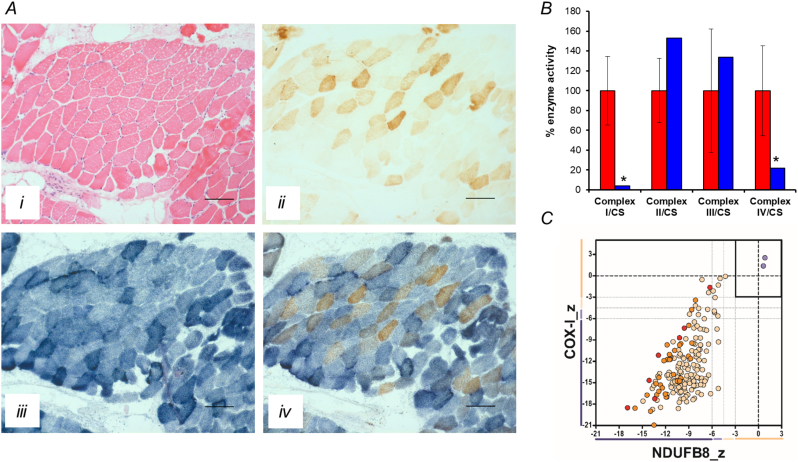
Histopathological and biochemical evaluation of skeletal muscle. (A) Histological and histochemical analyses of the patient's skeletal muscle biopsy showing hematoxylin and eosin (H&E) staining (i), cytochrome *c* oxidase (COX) histochemistry (ii), succinate dehydrogenase (SDH) histochemistry (iii) and sequential COX-SDH histochemistry (iv), highlighting the marked COX defect; scale bar = 100 μm. (B) The assessment of individual respiratory chain enzyme activities identified a severe, multiple OXPHOS deficiency affecting complexes I and IV in patient muscle (blue bars) compared to controls (red bars); mean enzyme activities shown for muscle controls (*n* = 25) are set at 100%. (C) Quadruple immunofluorescence analysis of NDUFB8 (complex I) and COXI (complex IV) mitochondrial subunits, confirming a multiple OXPHOS defect. Each dot represents the measurement from an individual muscle fibre, colour co-ordinated according to its mitochondrial mass (low = blue, normal = beige, high = orange, very high = red). Gray dashed lines represent SD limits for classification of the fibres. Lines next to x- and y-axes represent the levels of NDUFB8 and COXI: beige = normal (> − 3), light beige = intermediate positive (−3 to −4.5), light purple = intermediate negative (−4.5 to −6), purple = deficient (<−6). Bold dashed lines represent the mean expression level of normal fibres. (For interpretation of the references to colour in this figure legend, the reader is referred to the web version of this article.)

Muscle biopsy analysis showed normal fibre size following H&E staining, with evidence of fat deposition between fibres and fascicles (Fig. 2A). In addition, we noted remarkable mitochondrial histochemical abnormalities characterized by a mosaic pattern of COX-deficiency affecting >80% of all fibres on both the individual enzyme reaction and the sequential COX-SDH reaction and subsarcolemmal mitochondrial accumulation (ragged-blue fibres affecting ~5% of the total biopsy) on the individual SDH reaction, consistent with a mitochondrial aetiology (Fig. 2A). The assessment of mitochondrial respiratory chain enzyme activities in a frozen skeletal muscle homogenate revealed evidence of severe, multiple respiratory chain defects involving complexes I and IV with sparing of complex II activity, suggestive of a generalised defect of mitochondrial translation (Fig. 2B). In agreement with this, quadruple OXPHOS immunofluorescence confirmed the presence of many muscle fibres lacking both complex I (NDUFB8) and complex IV (COX-1) expression, confirming a multiple respiratory chain defect (Fig. 2C).

### 3.4 Molecular genetic analyses

**Fig. 3:**
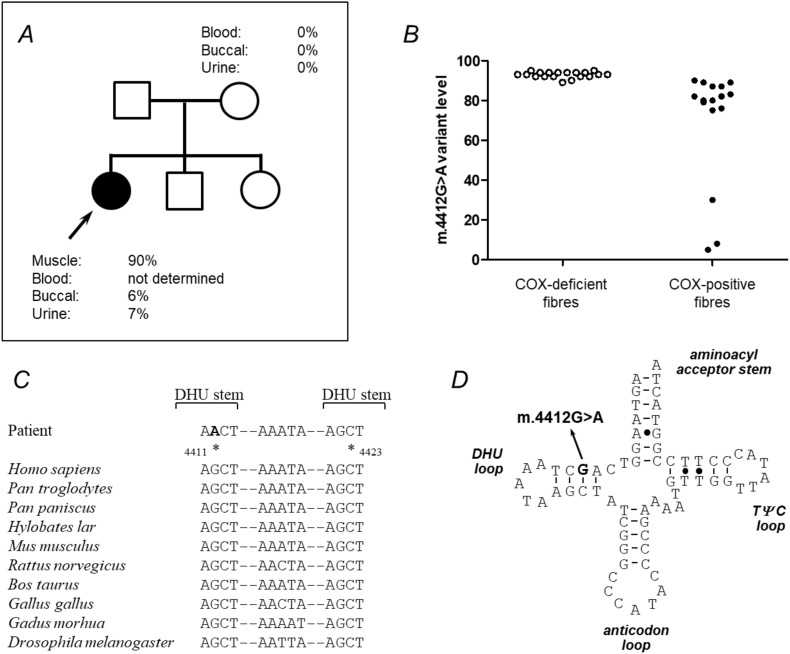
Mitochondrial DNA studies revealing a pathogenic m.4412A>G variant. (A) Family pedigree identifying the level of the novel *MT-TM* variant in the proband (indicated by an arrow), and the absence of this variant in all tissues tested in her mother. (B) Single fibre PCR analysis clearly shows a marked segregation of the m.4412A>G mutation with a biochemical defect in individual COX-deficient muscle fibres which harbour higher levels of mutation than COX-positive fibres (see text for details); each symbol represents data for one fibre. (C) Phylogenetic conservation of this region of the *MT-TM* gene sequence indicates the mutation affects an evolutionary conserved residue and base pair within the DHU stem, as further illustrated on the schematic representation of the mt-RNA^Met^ cloverleaf structure (D).

A peripheral blood leucocyte sample was screened for common pathogenic mtDNA variants but no abnormalities were detected. Sequencing of the complete mitochondrial genome revealed a novel m.4412G>A *MT-TM* variant at high levels of heteroplasmy (92% mutation load based on NGS reads) in skeletal muscle. Quantitative pyrosequencing confirmed high levels of heteroplasmy in skeletal muscle (90% mutation load), with much lower levels of heteroplasmy in buccal and urine (6% and 7% respectively) DNA samples. DNA samples obtained from the patient's mother showed no evidence of the novel m.4412G>A variant in buccal epithelia, urinary sediment or a blood sample implying the variant had likely arisen *de novo* during embryogenesis and not been maternally-inherited (Fig. 3A). Single muscle fibre analysis of individual COX-positive and COX-deficient fibres revealed a statistically-significant higher mutation load in COX-deficient fibres (92.89 ± 0.36% (*n* = 19 fibres)) than in COX-positive fibres (COX-positive fibres: 70.13 ± 7.12% (*n* = 16 fibres); *p* < .0001) confirming pathogenicity of the m.4412G>A variant (Fig. 3B).

## 4 Discussion

We described a young female who carried a novel variant, m.4412A>G in the *MT-TM* gene, resulting in the childhood onset of mitochondrial disease with multi-system involvement that included prolonged seizures, hypotonia, fatigue, proximal muscle weakness, retinopathy and short stature secondary to growth failure. Gross motor dysfunction was gradually progressive with significant deterioration of her gait and coordination, which appear to coincide with the development of bilateral signal change in the head of caudate on cranial MRI at age 13 years. Although she had some degree of learning difficulties following her first seizure, bilateral basal ganglia changes and elevated CSF lactate levels, this girl did not have the typical stepwise neuro-regression to fulfil the criteria for a diagnosis of Leigh syndrome, the commonest syndromic presentation of paediatric mitochondrial disease.

We believe that the novel m.4412A>G *MT-TM* gene variant can be classed as pathogenic for several reasons. First, this variant was not listed on a publically-accessible mitochondrial genome database (http://www.mitomap.org/) which contains Genbank frequency data from 47,412 human mitochondrial DNA sequence or our own in-house database of >1900 mtDNA sequences. Second, the highest level of mutation was found in clinically-affected skeletal muscle, with lower levels in blood. Third, this mutation appeared to have arisen *de novo* and was not detected in the patient's asymptomatic mother. Furthermore, this variant was located within the D-stem of the mt-tRNA^Met^ molecule disrupting an evolutionary-conserved Watson-Crick base pair within this stem which alters tRNA secondary structure and impedes its function (Fig. 3C and D). In addition, we demonstrated histochemical evidence of marked Complex I and Complex IV deficiency in muscle as evidence of a generalised defect of translation, consistent with a mt-tRNA defect. The most important evidence of pathogenicity is the single fibre segregation study which established that this variant segregates with the COX histochemical defect in her skeletal muscle tissue, thus confirming its pathogenicity. Using the validated scoring system(Yarham et al., 2011) which encompasses the biochemical and molecular genetics to assign pathogenicity, this m.4412A>G base substitutions had also reached the required threshold (a score of 13 out of 20) to distinguish itself from polymorphic changes in the mt-tRNA.

Our affected child has the earliest disease onset compared to the other reported cases of m.4409T>C, m.4403T>C, m.4440G>A, m.4437C>T and m.4450G>A variants within the *MT-TM* gene; the clinical phenotypes of these reported cases are summarised in Table 1. Most of these patients including ours, had progressive myopathy with elevated creatine kinase(Vissing et al., 1998; Peverelli et al., 2014; Scarpelli et al., 2018; Born et al., 2015). Interestingly, our young patient also had the highest heteroplasmy level in the muscle, suggesting that this extremely high mutation load might have predisposed her to an earlier onset of disease than the other reported patients who only became symptomatic later. Apart from the muscle phenotype, epileptic seizure have also been reported associated with the m.4437C>T and m.4450G>A variants(Tang et al., 2013; Kuwajima et al., 2019). One patient with m.4450G>A presented with a” stroke-like” episode, along with unilateral occipital lobe and bilateral basal ganglia changes(Kuwajima et al., 2019). Short stature, as documented in our patient, was observed in another patient(Vissing et al., 1998). As far as we know, none of the reported patients with pathogenic *MT-TM* variants had status epilepticus and pigmentary retinopathy as described in our patient. This paediatric case expands the current phenotypic spectrum of disease related to *MT-TM* mutations, as well as our understanding of pathogenic mt-tRNA gene variants and their impact across both paediatric and adult specialities.

**Table 1:** A summary of patients reported in the literature with pathogenic variants in the *MT-TM* gene. n.d. = not determined.

*MT-TM* gene variant	m.4412G>A	m.4403T>C	m.4409T>C	m.4437C>T	m.4440G>A	m.4450G>A		
Number of cases	1	1	1	1	1	3		
Literature citation	Current case (2019)	Peverelli et al. (2014)	Vissing et al. (1998)	Tang et al. (2013)	Scarpelli et al. (2018)	Lombes et al. (1998)	Born et al. (2015)	Kuwajima et al. (2019)

Clinical presentation								
Age at onset	2 years	56 years	10 years	13 years	53 years	66 years	10 years	8 years
Sex	Female	Female	Female	Female	Male	Male	Female	Female
Myopathy	Yes	Yes	Yes	Yes	Yes	No	Yes	No
Epilepsy	Yes	No	No	Yes	No	n.d.	No	Yes
Retinopathy	Yes	No	No	n.d.	n.d.	n.d.	No	n.d.
Short stature	Yes	n.d.	Yes	n.d.	n.d.	n.d.	n.d.	n.d.
Other features	micrognathia, hypotonia	n.d.	non-specific headache	hearing loss, hypotonia	n.d.	splenic lymphoma	intellectual disability	“stroke-like” episode

Investigations								
Creatine kinase levels	316 U/L	600–700 U/L	Raised	n.d	Elevated 5-fold	n.d.	Normal	38 U/L
Serum lactate levels	7.5 mmol/L	4.5 mmol/L	2.8 mmol/L	Noted to be elevated	n.d.	n.d.	5.3 mmol/L	11.6 mmol/L
CSF lactate levels	6.9 mmol/L	n.d.	n.d.	n.d.	n.d.	n.d.	n.d.	7.2 mmol/L
Neuroimaging results	Bilateral caudate head changes	Moderate global cortical atrophy	Normal	n.d.	n.d.	n.d.	Normal	Left occipital lobe & bilateral basal ganglia changes

## 5 Conclusion

In summary, we report a novel m.4412A>G variant in the *MT-TM* gene presenting with childhood-onset mitochondrial disease characterized by myopathy, prolonged seizures, retinopathy, short stature, lactic acidosis and bilateral basal ganglia changes. This mtDNA novel variant adds to the list of pathogenic *MT-TM* variants and illustrates the importance of a diagnostic muscle biopsy in demonstrating histocytochemical mitochondrial abnormalities and supporting single-fibre segregation studies.
